# Variations in DNA Methylation Are Landmarks of Freshwater Adaptation in Three-Spined Sticklebacks

**DOI:** 10.3390/ijms27104265

**Published:** 2026-05-11

**Authors:** Alexey Starshin, Alexandr Mazur, Nikolai Mugue, Daria Kaplun, Artemiy Golden, Ekaterina Khrameeva, Egor Prokhortchouk

**Affiliations:** 1 Institute of Bioengineering, Federal Research Center of Biotechnology, 117312 Moscow, Russia; starshin.alexey@gmail.com (A.S.); mazur.am@gmail.com (A.M.); 2 Scientific Center of Genetics and Life Sciences, Sirius University of Science and Technology, 354340 Sochi, Russia; 3 Koltzov Institute of Developmental Biology RAS, 119334 Moscow, Russia; 4 Center for Bio- and Medical Technologies, The Territory of the Skolkovo Innovation Center, 121205 Moscow, Russia

**Keywords:** sticklebacks, DNA methylation entropy, epigenetic variation, adaptation

## Abstract

Understanding the phenotypic consequences of epigenetic variation and its role in adaptation remains a central challenge in evolutionary biology. Marine and freshwater sticklebacks provide a powerful system to study the interplay between genetic and epigenetic components of phenotypic plasticity that enables colonization of contrasting salinity habitats. Here, we used whole-genome bisulfite sequencing (WGBS) to characterize DNA methylation entropy—a measure of epigenetic stochasticity—in gill tissue from marine and freshwater ecotypes. We found that freshwater sticklebacks exhibit elevated methylation entropy in divergence islands (DIs), genomic regions known as hotspots of genetic divergence between marine and freshwater populations. Within DIs, we identified a subset of genes exhibiting concurrent increases in methylation entropy and transcriptional variance, including osmoregulatory candidates involved in growth modulation, cytoskeletal reorganization, metabolism, and extracellular matrix remodeling. Their linked variability suggests that they may act as “adaptation capacitors” facilitating phenotypic plasticity during salinity transitions. Exploratory enrichment analysis further revealed overrepresented epigenetic regulators within DIs, such as DNA demethylase TET1 and chromatin remodelers ARID5B and BPTF, indicating a potential regulatory basis by which these factors may convert genetic variation into epigenetic diversity. Collectively, our findings demonstrate that DIs are focal points of both genetic divergence and epigenetic heterogeneity, consistent with a model in which DIs may act as multi-layered genomic regions associated with adaptive responses to salinity change.

## 1. Introduction

Any kind of phenotype in complex organisms arises from the interplay of two primary forces: genetic and environmental. Specifically, external conditions exert their influence on the organism by modulating genome function. In vertebrates, gene regulation is orchestrated through a multi-layered system, encompassing transcription factor networks, the dynamic remodeling of chromatin architecture and accessibility, and key epigenetic mechanisms such as DNA methylation and histone modifications. While standing genetic variation—the pool of pre-existing alleles in a population—is the primary source for natural selection, epigenetic states can facilitate rapid phenotypic responses to environmental change. Furthermore, beyond its regulatory role, DNA methylation can influence evolutionary trajectories by introducing a mutation bias at methylated CpG sites [1]. Similar to the role of genetic diversity in increasing the chances of a population to survive and reproduce, the variability of epigenetic marks may provide an additional reservoir of adaptive potential. For instance, cancer cells exhibit increased variability of DNA methylation in genomic regions known as Variably Methylated Regions (VMRs) [2], a phenomenon not observed in adjacent normal somatic cells. Remarkably, these VMRs are situated at critical developmental loci, including those implicated in axial pattern formation, neurogenesis, immune system development, and gut development [2]. Subsequent investigations have revealed that the boundaries between regions with high and low methylation entropy mainly coincide with borders between topologically associating domains (TADs) [3].

Biologists are now discussing how environmental changes are translated into epigenetic variations of both somatic and germ cells, what molecular mechanisms enable the transmission of epigenetic information through sexual reproduction, and how genetic selection influences epiallele frequency and diversity. For evolutionary biology, the phenotypic consequences of non-genetic inheritance (NGI) and their potential contribution to adaptation and diversification are pressing issues. Parental exposure to altered environmental conditions has been demonstrated to influence offspring morphology [4], physiology [5], behavior [6], longevity [7], and disease [8]. In contexts of adaptation, such exposure can enhance offspring fitness [5,9,10]. A growing body of work suggests that epigenetics can contribute to adaptation at the population level, maintaining phenotypic variation across generations [11,12,13]. The overall significance of epigenetics for evolutionary processes depends on the relative importance of NGI and genetic variation in creating phenotypic diversity [14]. Indeed, environmental variation can mediate the evolution of NGI regulation in roundworms [15]. However, data on the variability of NGI and its genetic basis from natural populations and from vertebrates is scarce.

Several groups have studied the genetic and epigenetic adaptation of marine three-spined sticklebacks to freshwater [16,17,18,19]. The process of adaptation began approximately 700 years ago when fish from the White Sea became isolated in Mashinnoe Lake due to the steady glacio-isostatic rise of the coast. Since then, the freshwater morph has adapted to low salinity, accompanied by shifts in key phenotypic traits. These adaptations are reflected in the genomes: marine and freshwater forms are differentiated at dozens of divergence islands (DIs), which show significantly divergent allele frequencies [17,20]. At the epigenetic level, the morphs are distinguished by multiple differentially methylated regions [18]. Intriguingly, these epigenetic changes appear to be linked to genomic evolution; Ord et al. demonstrated that sites which lost methylation in freshwater sticklebacks exhibited elevated nucleotide diversity [21], suggesting a potential mechanism by which epigenetic erosion can facilitate genetic diversification in a new environment.

Here, we employed whole-genome bisulfite sequencing (WGBS), which provides per-allele information with single-nucleotide resolution to characterize the stochasticity of DNA methylation (i.e., methylation entropy) in marine and freshwater sticklebacks. DNA methylation entropy was studied both at the whole-genome level and specifically within DIs. Altogether, we aimed to find an epigenetic component in the biology of stickleback adaptation to the altered environment with different salinity.

## 2. Results

### 2.1. DNA Methylation Entropy

**Figure 1:**
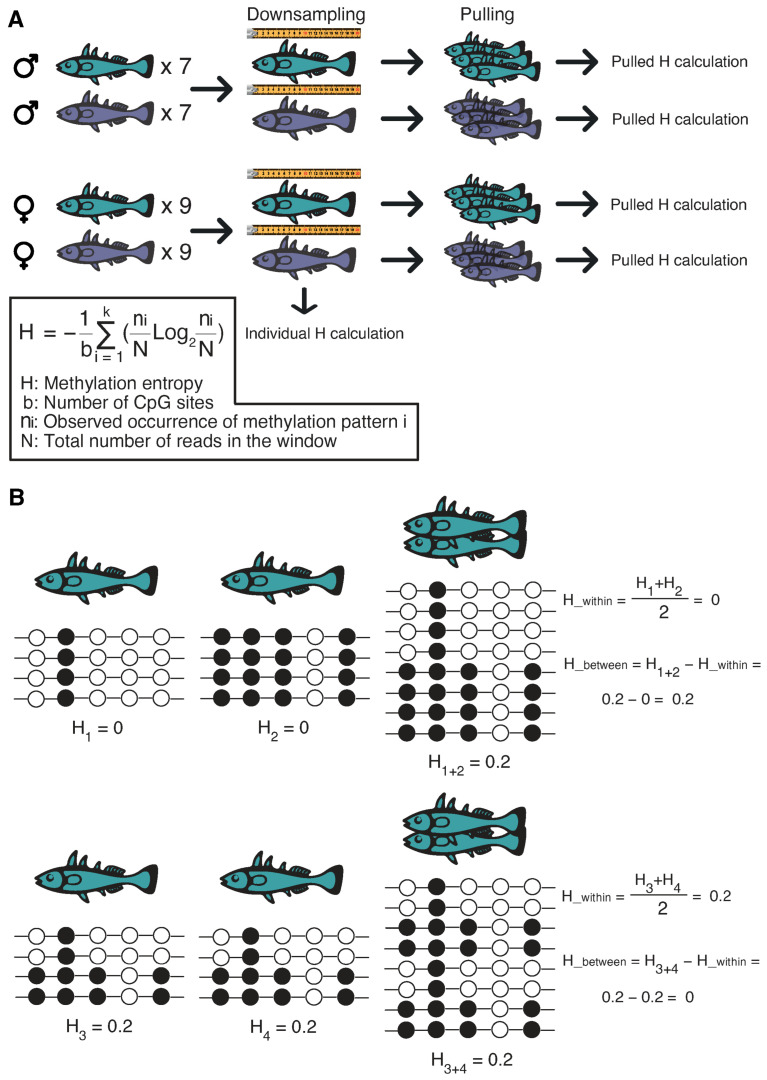
DNA methylation entropy. (**A**) Experimental design for DNA methylation analysis. Freshwater fish are denoted in green, marine fish in dark blue. (**B**) Illustration of methylation entropy calculation for a five-CpG window. The entropy value H was derived by considering the frequencies of all observed methylation patterns (epialleles) in a given window; the summation includes all k unique patterns (theoretically up to 32 for five CpGs). Black circles represent methylated CpG sites, and white circles represent unmethylated sites. In the first case, the total entropy can be explained by heterogeneity between samples. In the second case, the entropy is explained by heterogeneity within samples. For illustrative purposes, only extreme cases were taken: when all CpGs are methylated or all CpGs are unmethylated.

To explore the diversity of DNA methylation in stickleback populations, we examined 16 freshwater and 16 marine individuals, comprising 14 males and 18 females (Figure 1A). Given the critical impact of coverage on entropy estimates, we assessed the sequencing depth of each individual at a one-kilobase resolution (see Section 4). Next, we pooled downsampled individuals into four groups: Freshwater-Male, Marine-Male, Freshwater-Female, and Marine-Female. Methylation entropy was calculated both collectively for the four groups and individually per sample. Briefly, using a five-CpG sliding window, we calculated the frequency of each observed CpG state combination (epiallele frequency), and the methylation entropy was derived as a logarithmic function of these epiallele frequencies (a detailed description is provided in Section 4).

The choice of sliding window size reflects a balance between the sequencing depth, the distribution of window lengths across the genome, and combinatorial sufficiency. We selected a five-CpG window as a compromise between sufficient combinatorial complexity and relatively low coverage requirements (Figures S1–S5). We also calculated methylation entropy using four- and six-CpG windows and found that regardless of window size, our key findings remained consistent (Figures S6 and S7).

**Figure 2:**
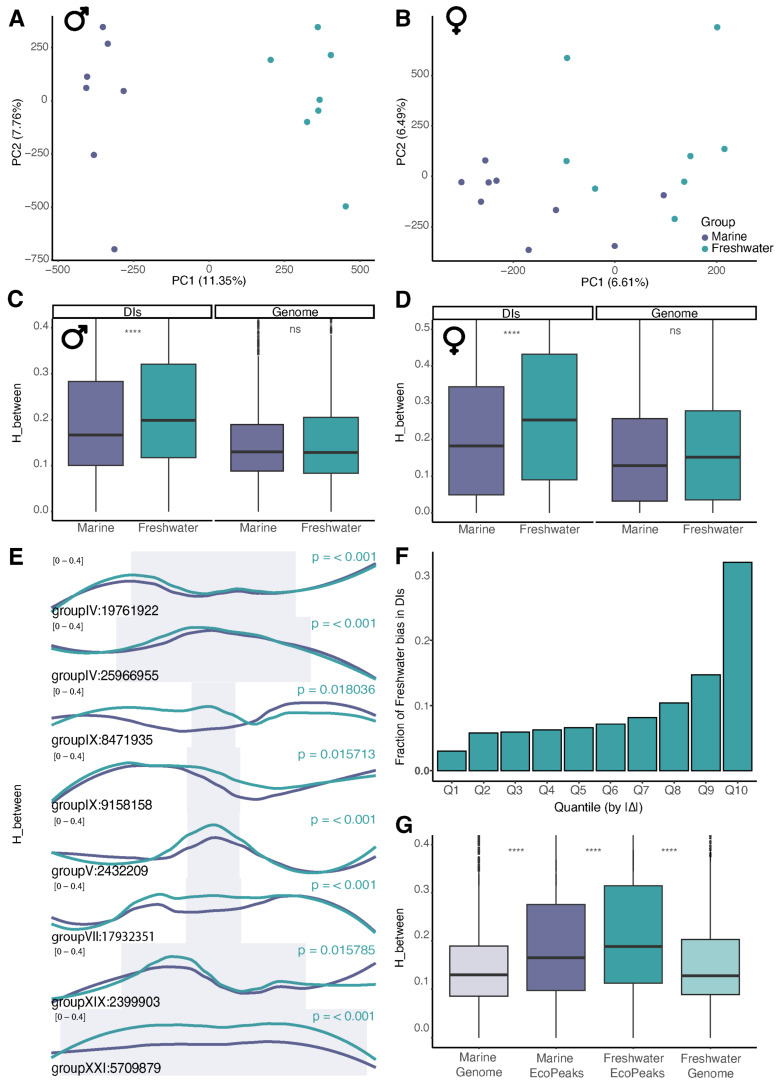
Elevated methylation entropy within divergence islands in freshwater fish. (**A**,**B**) PCA plots illustrating entropy variation among all male samples (**A**) and among all female samples (**B**). (**C**,**D**) Genome-wide differences in inter-sample methylation entropy and differences within divergence islands (DIs)—males (**C**) and females (**D**). (**E**) Inter-sample DNA methylation entropy of marine (dark purple) and freshwater (green) fish within illustrative divergence islands (DIs) and their 50 kb flanking regions. Each shown DI is individually scaled and adjusted to display the 50 kb flanking regions. DIs are highlighted by blue boxes. Genomic coordinates correspond to the Stickleback Feb. 2006 (Broad/gasAcu1) genome assembly. (**F**) Fraction of DIs with freshwater bias (Δ = Freshwater − Marine > 0) in each quantile of absolute entropy differences |Δ|. Quantiles are calculated across all data. (**G**) Inter-sample DNA methylation entropy in global specific EcoPeaks shows a similar effect to DIs. Significance in all panels is assessed by the Wilcoxon rank-sum test: ****—*p* < 0.0001, ns—not significant.

Focusing on population-level differences between marine and freshwater sticklebacks, we analyzed the entropy component specifically reflecting inter-individual variation (Figure 1B). To maintain methodological consistency with our downsampling approach (which created separate pools for males and females) and to avoid confounding effects of sex, we compared male and female sample groups independently when assessing environmental influences. Principal component analysis (PCA) indicated a clear trend of environmental separation, highlighting salinity as a key differentiating factor (Figure 2A).

However, initial genome-wide analysis showed no significant entropy differences between the two ecological groups (Figure 2B), indicating a high degree of conservation in global epigenetic regulation.

### 2.2. Elevated Methylation Entropy Within Divergence Islands in Freshwater Fish

Genetic variations trace the difference between freshwater and marine populations. Previous studies have indicated that, for these populations, the whole-genome fixation index (Fst) is significantly lower than the Fst within divergence islands (DIs) [20]. These findings suggest that the primary genetic variations associated with environmental adaptations are concentrated within marker SNPs of the DIs. To investigate whether DNA methylation entropy also exhibits differential patterns within DIs, we examined these DIs along with their flanking genomic regions of +/− 50 kb. Our analysis reveals that, in freshwater fish, DIs tend to exhibit higher DNA methylation entropy compared to marine ones (Figure 2B,C).

To further dissect the asymmetry in entropy differences between freshwater and marine populations, we analyzed the distribution of DIs across quantiles of absolute H_between divergence. While earlier visualizations (boxplots and LOESS curves) revealed a systemic shift toward higher entropy in freshwater populations, quantile decomposition allowed us to assess whether this bias is concentrated in loci with the largest divergences.

We partitioned all five-CpG windows into 10 quantiles based on |ΔH_between| (Freshwater-Marine), where Q10 represents the top 10% of divergences, and calculated the proportion of freshwater-preferring DIs (ΔH_between > 0) within each quantile. Interestingly, freshwater-preferring DIs were overrepresented in the highest quantiles (Figure 2D): for instance, Q9 + Q10 contained ~50% of all such DIs. This pattern suggests that the most divergent loci disproportionately contribute to the freshwater shift, possibly reflecting selective pressures or mutational biases specific to freshwater adaptation.

Since divergence islands (DIs) are characterized by altered allele frequencies between marine and freshwater populations, we investigated the possibility that elevated methylation entropy in these regions could be driven by underlying C→T mutations rather than genuine epigenetic variation. Cytosine-to-thymine transitions in CpG contexts are indistinguishable from true methylation changes in bisulfite sequencing data, as both result in C→T conversions.

To control for this potential confounder, we leveraged whole-genome sequencing (WGS) data from Terekhanova et al. (2019), calculating entropy specifically for C→T transitions using the same methodology applied to methylation data [20]. This approach allowed us to distinguish entropy attributable to genetic mutations from that arising from bona fide methylation variation.

The C→T transition entropy did not differ significantly between marine and freshwater populations within DIs (Figure S8), while the overall level of such entropy was relatively low. This confirms that the increased methylation entropy in freshwater DIs reflects genuine epigenetic variability rather than an artifactual signal from mutation-derived C→T substitutions.

In addition to the DIs presented, we investigated the distribution of methylation entropy in other known stickleback loci that drive the adaptation process. For instance, the study by Roberts Kingman et al. introduced a newly defined set of EcoPeaks (haplotypes observed to convergently evolve in freshwater conditions) and TempoPeaks (haplotypes that evolve in contemporarily evolving ponds) [22]. The peaks were identified using different geographic sets of samples (all samples—global, northeast Pacific populations and populations of the Atlantic coasts) and two levels of stringency (1% FDR—specific, 5% FDR—sensitive). These regions are conceptually related but not identical to the DIs used in our primary analysis, as they were defined in a different study using different population sets and criteria for identifying loci associated with freshwater adaptation. We therefore examined them as an external comparative framework to assess whether elevated methylation entropy is a general feature of adaptation-associated loci or is more specifically concentrated within the DIs analyzed here.

Global specific EcoPeaks exhibited methylation entropy patterns consistent with those observed in divergence islands (DIs) (Figure 2E). Pacific specific EcoPeaks showed a similar trend, though the effect was less pronounced (Figure S9). However, across all sensitive EcoPeaks and TempoPeaks, we detected no statistically significant differences in methylation entropy (Figures S10–S13).

In summary, our findings suggest that DIs represent a significant fraction of genomic regions characterized by increased methylation entropy in freshwater fish. This may represent a distinct epigenetic hallmark of sticklebacks’ adaptation to water salinity conditions.

### 2.3. Methylation Entropy May Shape Transcriptional Outputs in Divergence Islands Through Context-Dependent Mechanisms

**Figure 3:**
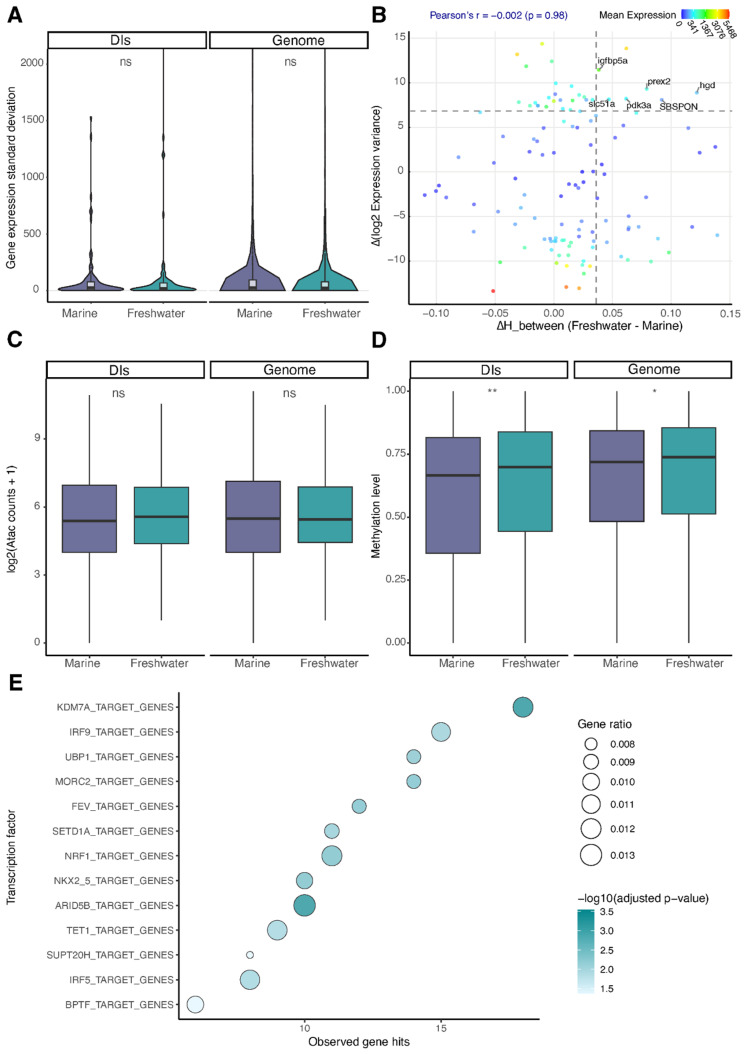
Multi-omics profiling of divergence islands (DIs). (**A**) Inter-individual expression SD (DESeq2-normalized counts; four biological replicates per ecotype). (**B**) DNA methylation entropy vs. transcriptional variability within DIs. Each point represents a gene promoter; colors indicate mean expression level among all samples; dashed lines indicate the thresholds used to highlight genes of potential interest. (**C**) Chromatin accessibility in the whole genome and DI regions (two biological replicates per ecotype). Statistical comparisons based on normalized counts; values are log2-transformed for visual representation. (**D**) CpG methylation levels (β-values) in the whole genome and DI regions. (**E**) Top potential freshwater-specific gene regulators enriched in DIs. Significance in all panels is assessed using the Wilcoxon rank-sum test: *—*p* < 0.05, **—*p* < 0.01, ns—not significant.

To assess the potential regulatory consequences of differential methylation entropy, we performed integrated analysis of transcriptional and chromatin profiles. First, we examined expression patterns of 98 genes located within freshwater-associated DIs using bulk RNA-seq data from [18]. Neither the median expression levels (Figure S14) nor expression standard deviation (Figure 3A) differed significantly between marine and freshwater ecotypes. Intercellular variance according to our scRNA-seq data also showed no statistically significant differences (Figure S15).

To investigate the potential functional links between epigenetic and transcriptional variability, we analyzed the promoter regions (1000 bp upstream TSS) of genes located within divergence islands (DIs). For each promoter, we calculated: (1) the mean difference in methylation entropy between freshwater and marine ecotypes, and (2) the difference in expression variance across individuals.

While genome-wide analysis did not detect a robust association between these measures in the present dataset (Pearson’s r = −0.002, *p* = 0.98), we identified a small subset of genes in the top quartile for both ΔHb and Δvariance that may represent exploratory candidates for freshwater adaptation (Figure 3B). Notably, these included the insulin-like growth factor binding protein (*igfbp5a*), potentially modulating growth during energetically demanding osmotic shifts [23]; *prex2* (Rac activator), which could facilitate the adaptive cytoskeletal reorganization critical for cell volume regulation under salinity stress [24]; *slc51a* (bile acid transporter OSTα-OSTβ), possibly maintaining essential lipid metabolism and bile acid flux during freshwater transition [25]; metabolic regulators *pdk3a* and *hgd*, potentially supporting the hypoxia response and detoxification pathways, respectively, under osmotic stress [26,27]; and *sbspon*—the extracellular matrix protein SBSPON, which may contribute to the structural remodeling of osmoregulatory tissues like gills.

Single-cell ATAC-seq analysis of pooled cells from both ecotypes revealed no systematic differences in chromatin accessibility within DI regions (Figure 3C).

Comparative analysis of whole-genome DNA methylation revealed significant differences between marine and freshwater populations (Figure 3D). This effect was more pronounced in divergence islands (Figure S16), though the magnitude of differentiation remained substantially smaller than that observed for methylation entropy. The consistent direction of changes (freshwater-biased) across both metrics suggests two parallel layers of regulatory evolution in DIs. However, the disproportionate increase in entropy implies stronger selection on methylation stochasticity than on mean methylation levels per se in these genomic regions.

### 2.4. Human Ortholog Analysis Suggests a Potential Regulatory Basis for DI-Associated Entropy

To explore possible regulatory mechanisms associated with elevated methylation entropy in freshwater DIs, we performed an exploratory enrichment analysis of DI-associated gene orthologs using rGREAT and the msigdb:C3 regulatory motif database. Because regulatory annotations for fish remain limited, these results should be interpreted as indirect, cross-species inferences. This approach revealed significant enrichment (FDR < 0.05) for transcription factors and chromatin remodelers including: DNA demethylases (TET1), histone modifiers (KDM7A and SETD1A), and chromatin remodelers (BPTF, MORC2, and ARID5B) (Figure 3E, Table S1). While these associations derive from human regulatory annotations, the consistent pattern across multiple epigenetic layers (DNA methylation, histone modification, and chromatin remodeling) suggests that DI genes may participate in coordinated regulatory processes.

## 3. Discussion

The three-spined sticklebacks have been a focus of evolutionary biologists since the late 1960s [28]. These small, easily accessible, and polymorphic fish were available to scientists in America, Europe, and Asia. Sticklebacks became popular as a model for studying various aspects of adaptation: the role of a diet in explaining differences between benthic and limnetic morphs [29]; body shape changes between stream and lake fish [30]; and marine and freshwater populations [16,17]. In all these cases, genetics was an obvious field of science that formed a basis for measurable characteristics to describe morphs. However, diet, water flow, and water salinity also have a strong environmental component that affects not only the natural choice of best genotypes but also shapes favorable epigenetic landscapes that facilitate the survival, growth, and reproduction of fish in particular surrounding circumstances. Given the incomparably longer time required for genetic adaptation, epigenetic mechanisms are preferable for facilitating rapid changes in fitted traits. However, epigenetic landscapes such as DNA methylation cooperate with genotypes: upon activation of adaptive gene reprogramming, some genotypes may be more or less favorable to gene functioning, and vice versa, epigenetic changes may compensate for unfavorable genotypes. Moreover, epigenetics produces an additional level of within-population diversity, resulting in more adaptive power of a species. Accordingly, we focused on the variability in the degree of DNA methylation in this work. Beyond genetic diversity, epigenetic dispersion represents an additional population characteristic that may reflect its capacity for adaptation at the level of gene regulation or environmental variability.

Our study reveals that elevated DNA methylation entropy in freshwater sticklebacks is not a genome-wide phenomenon, but specifically localized to divergence islands (DIs)—genomic regions previously identified as hotspots of genetic differentiation between marine and freshwater ecotypes. This spatial restriction of epigenetic variability is particularly striking given that approximately half of all freshwater DIs fall within the top two quantiles of entropy difference between freshwater and marine ecotypes, suggesting these regions may represent distinct epigenetic as well as genetic landscapes. The concentration of both genetic (Fst) and epigenetic divergence in DIs reinforces the role of these regions as evolutionary hotspots, consistent with previous reports of sharp linkage disequilibrium (LD) peaks in marine populations compared to broader LD patterns in freshwater-adapted fish [20]. Interestingly, this epigenetic divergence appears to be directed opposite to the genetic pattern: with reduced genetic diversity within DIs in freshwater fish, we observe higher epiallelic diversity. This inverse relationship may indicate a functional compensation for the loss of genetic variation through epigenetics. While our study cannot definitively resolve whether DI-associated entropy is currently under selection or represents a neutral evolutionary signature, the consistent localization of both genetic and epigenetic divergence to these regions suggests they remain important genomic arenas for adaptive evolution.

The relationship between DNA methylation entropy and gene expression within divergence islands (DIs) remains to be elucidated. The overall absence of correlation between promoter methylation entropy and transcriptional variance across DIs suggests these epigenetic changes may operate primarily through distal regulatory mechanisms rather than proximal promoter effects. The spatial concentration of entropy changes in these evolutionarily conserved regions—known to be enriched for developmental enhancers and boundary elements—suggests potential long-range regulatory consequences [3]. However, the limited statistical power inherent in our transcriptomic datasets—with only four individuals per ecotype for bulk RNA-seq and two individuals for single-cell RNA-seq—cannot be ruled out as a contributing factor to the lack of observed association.

We identify a set of hypothetical “adaptation capacitors”—loci where coupled increases in methylation entropy and expression variance (e.g., in igfbp5a, prex2, slc51a, pdk3a, sbspon, and hgd) may potentially contribute to phenotypic plasticity. Although this group represents only ~3% of DI-associated transcripts, these genes should be regarded as preliminary candidates consistent with a possible mechanism of rapid osmoregulatory adaptation.

The insulin-like growth factor binding protein Igfbp5a, demonstrated to regulate growth in fish like grass carp [23], may be potentially relevant during the energetically demanding transition between marine and freshwater environments. By modulating IGF signaling, Igfbp5a might help balance somatic growth against the substantial metabolic costs of osmoregulation (e.g., ion pumping and tissue remodeling), thereby optimizing energy allocation for survival and fitness. Similarly, the Rac activator Prex2, shown to influence cellular signaling pathways upon mutation in human systems [24], emerges as a candidate for facilitating rapid cytoskeletal reorganization. Given the central role of the actin cytoskeleton in cell volume regulation—a fundamental response to osmotic stress—Prex2 could enable adaptive structural changes in key osmoregulatory cells, such as gill ionocytes, ensuring functional integrity under fluctuating salinity.

Metabolic and homeostatic support also appears to be important. The bile acid transporter Slc51a (OSTα-OSTβ), essential for steroid-derived molecule flux [25], may potentially contribute to maintaining lipid metabolism during salinity transitions. Efficient bile acid transport underpins lipid digestion and absorption; thus, Slc51a could safeguard the continuous energy supply required to fuel osmoregulatory processes. Complementing this, the metabolic regulator Pdk3a represents a hypothetical candidate link for enhancing cellular resilience. Research in bass indicates that modulating Pdk activity (including Pdk3) promotes oxidative phosphorylation, improving glucose utilization and reducing oxidative stress [26]. Consequently, Pdk3a might contribute to metabolic flexibility under osmotic stress, potentially mitigating hypoxia and oxidative damage while meeting heightened ATP demands. Furthermore, the enzyme encoded by *hgd*, crucial for preventing toxic homogentisic acid accumulation in humans [27], suggests a potential role in detoxification pathways. Efficient catabolism of tyrosine/phenylalanine derivatives by Hgd could help manage metabolic byproducts and oxidative stress exacerbated by the physiological upheaval of salinity change.

Finally, structural adaptation is implicated through the extracellular matrix (ECM) protein Sbspon. As ECM components are fundamental to tissue integrity and plasticity, Sbspon could contribute to the essential remodeling of osmoregulatory tissues like the gills. This remodeling, involving changes in ionocyte density, vascularization, and connective tissue, is critical for optimizing ion transport efficiency in the new osmotic environment, and Sbspon may hypothetically participate in structural remodeling or modulate cell–ECM interactions to facilitate this process.

Despite all the above assumptions, we cannot statistically exclude the possibility that the co-occurrence of these genes in high-entropy regions reflects random clustering rather than functional adaptation.

An alternative interpretation emerges from the evolutionary context of freshwater adaptation. The enrichment of epigenetic regulator orthologs (including TET1, ARID5B, and chromatin remodelers) among DI-associated genes is consistent with the possibility that these regions may be predisposed to methylation plasticity. In this light, elevated entropy could represent either a historical signature of selection for phenotypic capacitance during freshwater colonization or an ongoing mechanism to maintain regulatory diversity in genomic regions where genetic variation has become constrained by selective sweeps. The latter possibility is particularly intriguing given evidence that DNA methylation can differentially affect transcription factor binding—some factors are excluded by methylated CpGs, while others preferentially bind methylated sequences. Thus, methylation entropy could theoretically expand the repertoire of possible regulatory states in freshwater DIs without requiring genetic polymorphism. However, the cross-species nature of this analysis requires cautious interpretation and experimental validation in fish models.

An additional limitation of this study is that, although the marine and freshwater fish were sampled from geographically close locations, they originate from distinct natural habitats and may differ not only in salinity exposure but also in other ecological and population-genetic factors. Therefore, the observed epigenetic differences should be interpreted as ecotype-associated patterns in this marine–freshwater system rather than effects attributable exclusively to salinity.

Another limitation of the present study is that it was designed primarily to characterize DNA methylation entropy rather than to provide a comprehensive multi-omics analysis. We observed a distinct entropy-associated pattern in divergence islands and used the additional molecular layers mainly to explore possible explanations and implications of this effect. Accordingly, the main conclusions of the manuscript remain centered on methylation entropy.

Future studies combining chromatin conformation capture techniques (e.g., Hi-C) with functional genomics and environmental manipulations will be crucial to test whether this localized epigenetic variability within DIs manifests functional consequences under ecologically relevant conditions. Collectively, our findings establish divergence islands as critical hubs of both genetic and epigenetic variation in sticklebacks, suggesting they function as integrated genomic–epigenetic platforms enabling rapid environmental adaptation. This epigenetic dimension of diversity likely underpins the remarkable phenotypic plasticity observed in this model system.

## 4. Materials and Methods

### 4.1. Samples

Fish were collected in the Mashinnoe Lake (freshwater morph—7 males and 9 females; 66°17.749 N, 33°21.829 E; estimated age 700 years) and from the marine shore at White Sea Biological Station (marine morph—7 males and 9 females; 66°57.040 N, 33°10.400 E). Gills were cut with sterile scissors. Gills were thoroughly washed with chilled 1 × PBS and transferred to a Petri dish. The tissue was cut into small pieces with sterile scissors and washed twice with chilled 1 × PBS. The pellet was trypsinized with 200 μL TrypLE ™ Express Enzyme (Gibco, Grand Island, NY, USA) for 2 min. One ml of fetal bovine serum (FBS; Hyclone, Logan, UT, USA) was added to the cell suspension to inhibit trypsin activity. The cells were harvested by filtering the cell suspension through a filter (80 microns). The filtrate was centrifuged at 2000 rpm for 5 min. Fish collection and all subsequent experimental procedures were approved by the Ethics Committee for Animal Research of the Koltzov Institute of Developmental Biology RAS (Approval Code 47; 8 April 2021).

### 4.2. Isolation of Nuclei

Nuclei were isolated according to the 10x Genomics protocol for “Nuclei Isolation for Single Cell Multiome ATAC + Gene Expression Sequencing” available at https://www.10xgenomics.com/. The cells were washed 2 times with 1 × PBS + 0.04% BSA, and the number of cells was determined. Nuclei were isolated from 100,000–1,000,000 cells. Briefly, 100,000–1,000,000 cells were added to a 2 mL microcentrifuge tube. It was centrifuged at 300 rpm for 5 min at 4 °C. All supernatant was removed without destroying the cell sediment. Then, we added 100 μL of chilled lysis buffer (10 mM Tris-HCl (pH 7.4), 10 mM NaCl, 3 mM MgCl_2_, 0.1% Tween-20, 0.1% Nonidet P40 Substitute (if using Sigma (74385) (Kawasaki, Japan) 100% solution, prepare a 10% stock), 0.01% Digitonin (incubate at 65 °C to dissolve precipitate before use), 1% BSA, 1 mM DTT, 1 U/µL RNase inhibitor, Nuclease-free water), and incubated it for 3–5 min on ice. We next evaluated the efficiency of lysis using an automatic cell counter and added 1 mL of chilled wash buffer (10 mM Tris-HCl (pH 7.4), 10 mM NaCl, 3 mM MgCl2, 1% BSA, 0.1% Tween-20, 1 mM DTT, 1 U/µL RNase inhibitor, Nuclease-free water) to the lysed cells. It was centrifuged at 500 rpm for 5 min at 4 °C. The supernatant was removed without disturbing the pellet of the nuclei. Based on cell concentration and assuming ~50% of nuclei lost during cell lysis, we resuspended in a chilled diluted nuclei buffer (1XNuclei Buffer (20X), 1mM DTT, 1 U/µL RNase inhibitor, Nuclease-free water; Table S2). All work procedures were performed on ice. We determined the concentration of nuclei using an automatic cell counter (Thermo Fisher Scientific, Waltham, USA), and then immediately switched to Chromium Single Cell ATAC Reagent.

### 4.3. Genomic DNA Isolation

DNA was extracted from the gill with the DNeasy Blood and Tissue Kit (Qiagen, Hilden, Germany) according to the manual. DNA purity was checked with Nanodrop ND-1000 and the ratios A260/A280 and A260/A230 were more than 1.8 for all samples. DNA integrity was checked by 1% TAE gel electrophoresis and the band at more than 10 kb was observed for all samples.

### 4.4. Bisulfite Conversion and Whole-Genome Bisulfite Sequencing

A total of 1 μg of Stickleback genomic DNA was mixed with 10 ng lambda phage DNA and sheared with ultrasound to the average size of 150 bp. End-repair, dA tailing, and methylated adaptor ligation were performed with NebNext DNA UltraII kit (NEB). After adaptor ligation, libraries were bisulfite converted with EZ DNA Methylation Kits (ZYMO RESEARCH, Irvine, CA, USA) according to the manufacturer’s protocol. After conversion, the final libraries were amplified with NEBNext Q5U^®^ Master Mix (NEB). Library fragment size distribution was done on Bioanalyzer2100 (Agilent, Santa Clara, CA, USA) with the DNA High Sensitivity kit (Agilent). Library concentration was measured on Qbit 2.0 with Qubit dsDNA High Sensitivity (Thermo, Waltham, MA, USA) and sequenced with Illumina HiSeq1500 (Illumina, San Diego, CA, USA).

### 4.5. Single-Cell RNA Sequencing (scRNA-Seq)

Single-cell experiments in gill samples of two freshwater and two marine sticklebacks were performed using a 10× Chromium single cell 3′ v2 reagent kit by precisely following the manufacturer’s detailed protocol to construct 10× Genomics single-cell 3′ libraries. Library fragment size distribution was done on a Bioanalyzer2100 (Agilent) with the DNA High Sensitivity kit (Agilent). Library concentration was measured on Qbit 2.0 with Qubit dsDNA High Sensitivity (Thermo). Single-cell libraries were run using paired-end sequencing on the HiSeq1500 platform (Illumina) according to the manufacturer’s instructions.

### 4.6. ATAC-Seq Experiments

ATAC-seq experiments in gill samples of two freshwater and two marine sticklebacks were performed using a 10× Chromium Single Cell ATAC Library & Gel Bead Kit by precisely following the manufacturer’s detailed protocol to construct a 10× Single Cell ATAC Library. Library fragment size distribution was done on a Bioanalyzer2100 (Agilent) with the DNA High Sensitivity kit (Agilent). Library concentration was measured on Qbit 2.0 with Qubit dsDNA High Sensitivity (Thermo). The libraries were run using paired-end sequencing on the HiSeq1500 platform (Illumina) according to the Chromium Single Cell ATAC Reagent Kits User Guide.

### 4.7. WGBS Data Processing

Paired-end reads (100 bp) were processed with Trim Galore ver. 0.5.0 [31] to remove adapter sequences and trim bases with low quality scores (<20). Validated reads were aligned to Broad/gasAcu1 genome assembly with the Bismark software ver. 0.24.2 [32] (Table S3). Bisulfite conversion efficiency (>99%) was assessed using both the lambda phage and methylation of non-CpG context.

The genome was divided into windows, each with a length of 1 kilobase and a step of 1 kilobase. For each sample, the coverage values of the 1 kb windows were calculated using the *featureCounts* program [33]. All 14 male samples were combined into one group, and their minimum coverage value in each window was used for downsampling each male sample individually. A similar procedure was carried out for the 18 female samples. As a result, the samples were combined into four downsampled groups: Freshwater-Female, Saline-Female, Freshwater-Male, and Saline-Male. A total of 93 and 94% of the 5-CpG windows were covered by at least 32 fragments in the male and female groups (Figures S1–S4).

The methylation entropy for the five-CpG bins was calculated as follows: all CpGs in the genome are divided into sliding windows with a length of five and a step of one CpG. Reads overlapping the sliding window provide patterns of CpG states (epialleles); for each epiallele, its frequency is calculated as the ratio of the number of a given pattern to all available patterns. The products are then summed up according to the formula:

$$-\frac{1}{b}\sum_{i=1}^{k}\;\left(\frac{n_{i}}{N}\;*\;{log}_{2}\frac{n_{i}}{N}\right),$$

where *b* is the number of CpG sites (5), the summation runs over all *k* unique epialleles observed in the window (*i* = 1, 2, …, *k*), $n_{i}$ is the number of reads displaying the *i*-th methylation pattern, and N is the total number of reads covering the window. For a 5-CpG window, the maximum possible value of *k* is 32 (representing all $2^{5}$ combinations of methylated and unmethylated states).

### 4.8. Single-Cell RNA-Seq Data Processing

A total of 1,133,906,325 paired-end sequencing reads of scRNA-seq were processed using the publicly available 10× Genomics software—Cell Ranger v3.1.0 [34] (Table S4). The sparse expression matrix generated by the Cell Ranger analysis pipeline with the list of 21,474 cells was used as input to the Seurat software ver. 3.1 [35].

Seurat pipeline standard quality control steps were performed, and cells were filtered for nFeature_RNA > 100 and percent of mitochondrial genes < 2 (Figure S17). Doublet detection was performed with Scrublet [36]. The detected doublet rate was below 0.7% for all samples.

To account for technical variation, we performed cross-species integration. At the first step, for marine and freshwater samples separately, we performed normalization using “LogNormalize” with the scale factor of 10,000 and identified 2000 variable features. We then performed cross-species integration by finding corresponding anchors in marine and freshwater samples using 30 dimensions. We then computed 50 principal components on the integrated data.

### 4.9. ATAC-Seq Data Processing

A total of 444,039,620 paired-end sequencing reads from four samples were processed using the publicly available 10× Genomics software Cell Ranger ATAC v2.0 [37] (Table S5). The sparse open chromatin peaks matrix generated by the Cell Ranger ATAC analysis pipeline with the list of 42,569 cells was used as input to the Signac software ver. 1.2.1 [38]. Signac pipeline standard quality control and cell filtration steps were performed for each sample individually; the parameters for filtration for each sample are presented in Table S6. Next, peaks from all four samples were merged following the Signac default “merging objects” procedure. For this, a unified set of peaks for all samples was created using the GenomicRanges package ver. 1.60.0 reduce() approach, which merges the overlapping peaks to form a single one. The resulting matrix containing a unified set of peaks was used for further analysis.

## Data Availability

The raw sequencing reads are deposited at SRA under the BioProject PRJNA765182.

## Supplementary Materials

The following supporting information can be downloaded at https://www.mdpi.com/article/10.3390/ijms27104265/s1.
