# Supplementary material for: Variations in DNA Methylation Are Landmarks of Freshwater Adaptation in Three-Spined Sticklebacks
Source: Int J Mol Sci. 2026 May 11;27(10):4265. doi: 10.3390/ijms27104265 (PMC13207980; doi:10.3390/ijms27104265)
Supplement: Supplementary file 1 [file ijms-27-04265-s001.zip › Variations in DNA methylation are landmarks of freshwater adaptation in threespine sticklebacks_SUPPLEMENT_IJMS.pdf]

Supplementary Figures

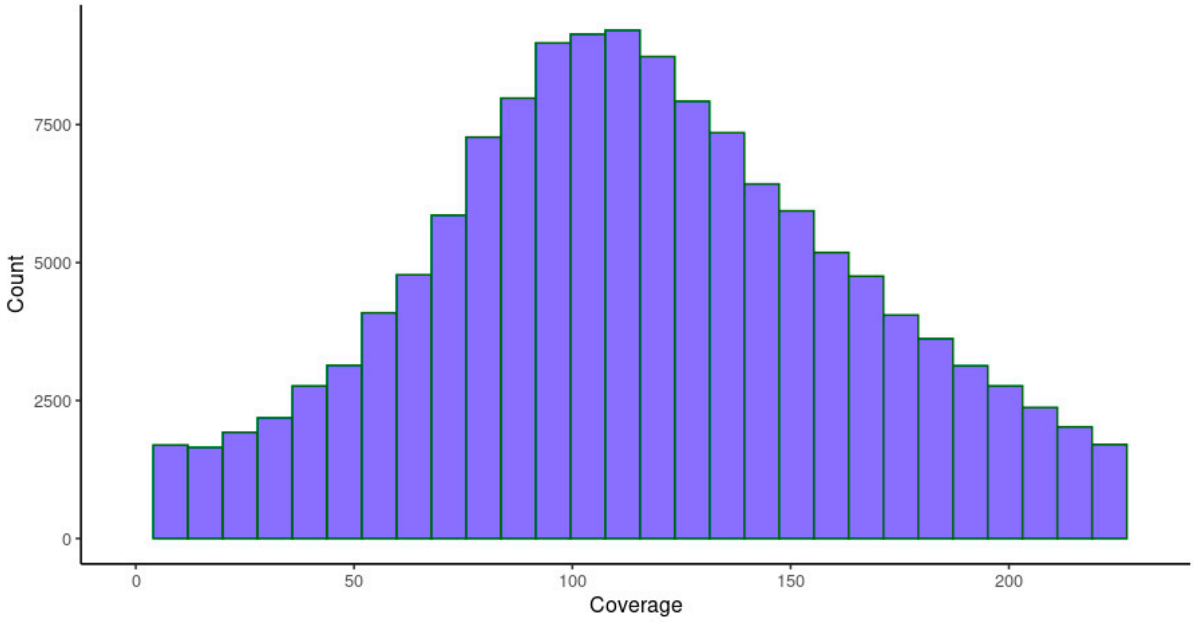

Figure S1. Distribution of coverage for 5-CpG windows in marine males.

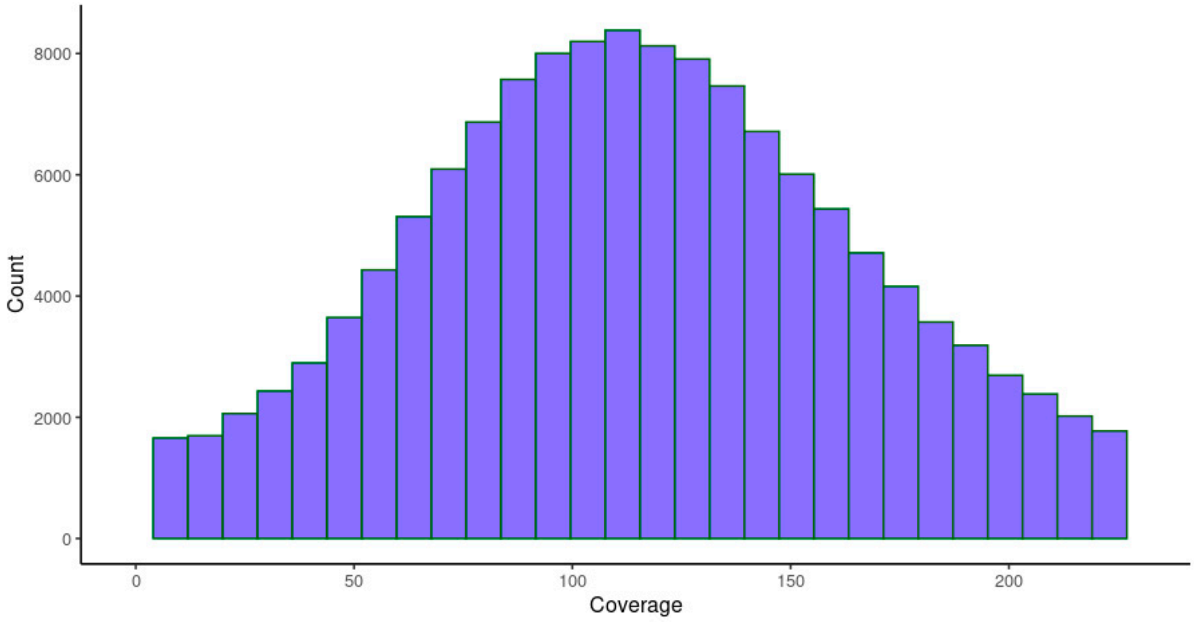

Figure S2. Distribution of coverage for 5-CpG windows in freshwater males.

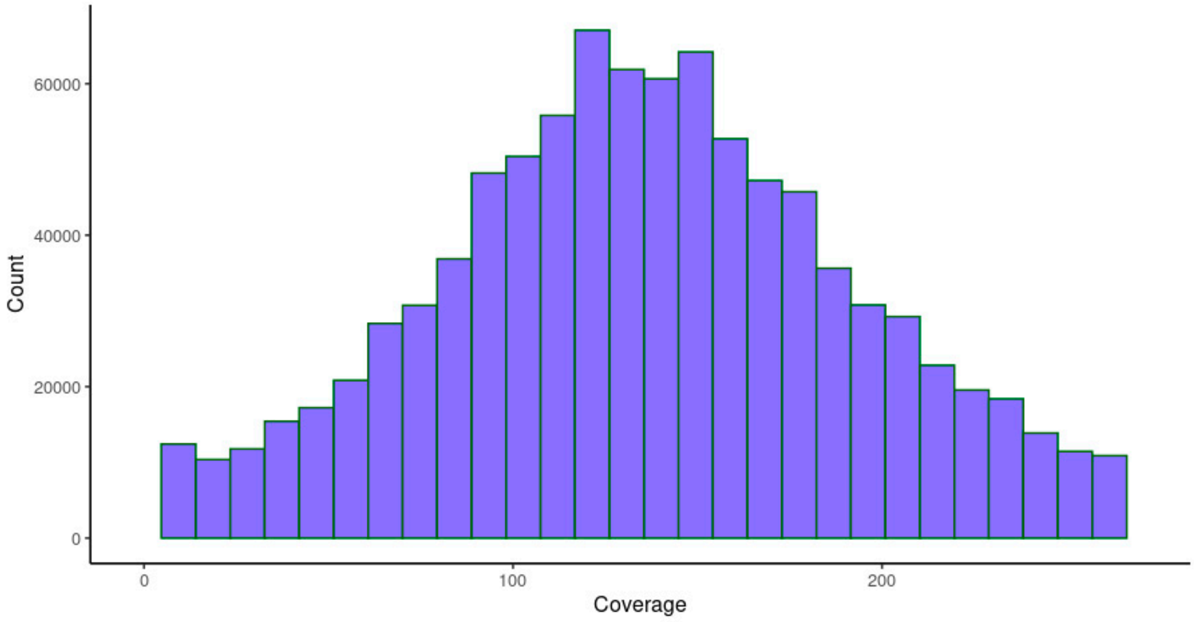

Figure S3. Distribution of coverage for 5-CpG windows in marine females.

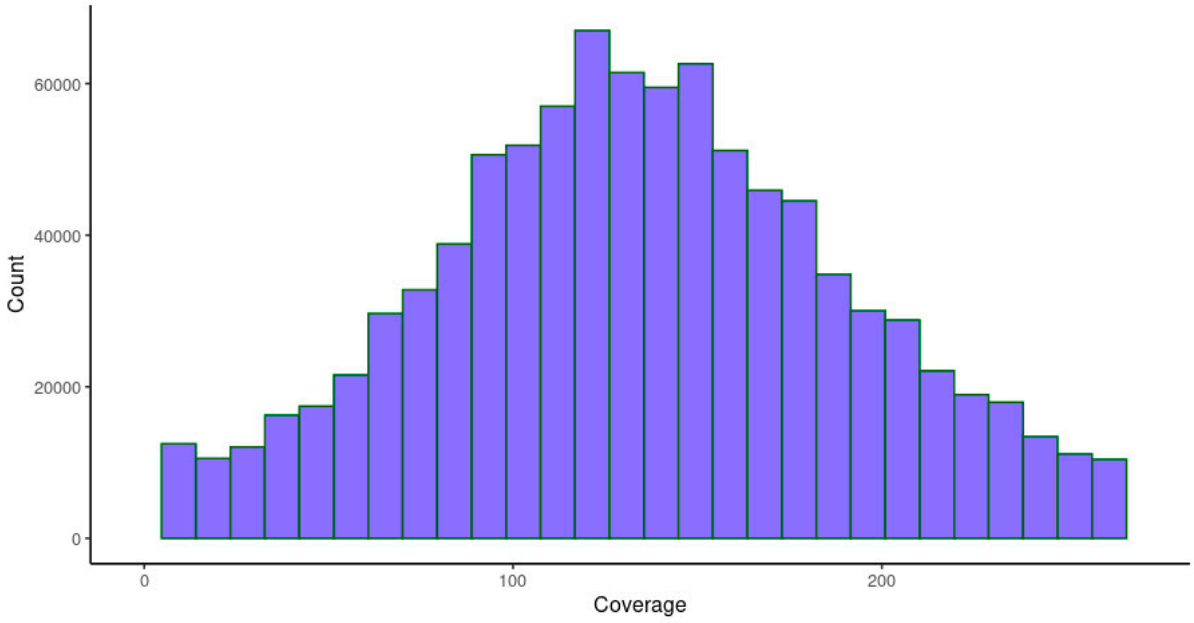

Figure S4. Distribution of coverage for 5-CpG windows in freshwater females.

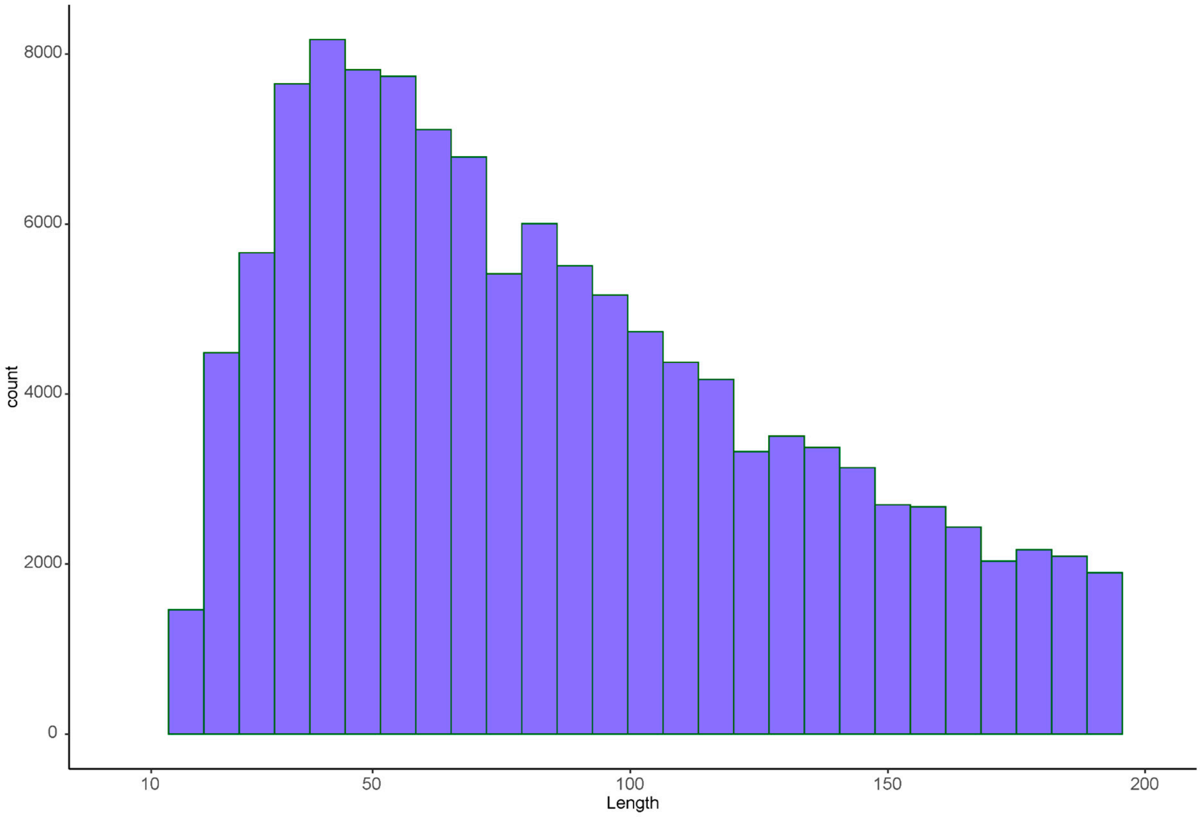

Figure S5. Genome-wide distribution of 5-CpG windows lengths.

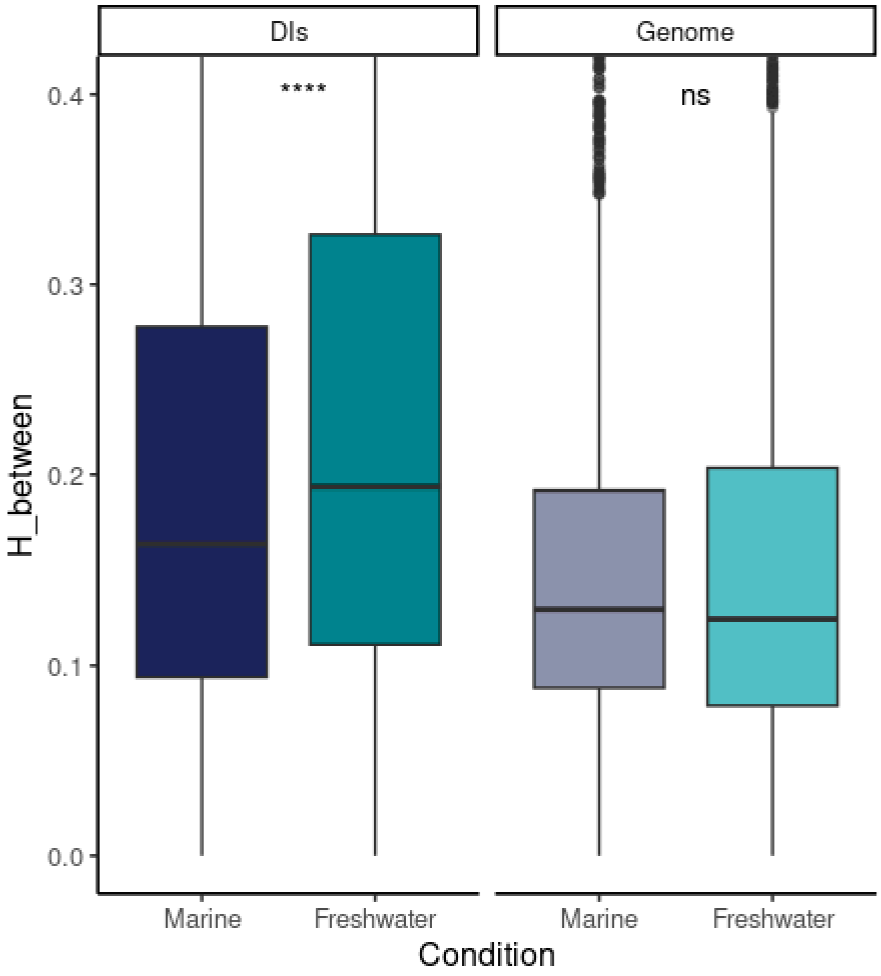

Figure S6. Genome-wide differences in inter-sample methylation entropy and differences within divergence islands (DIs); 4-CpG windows.

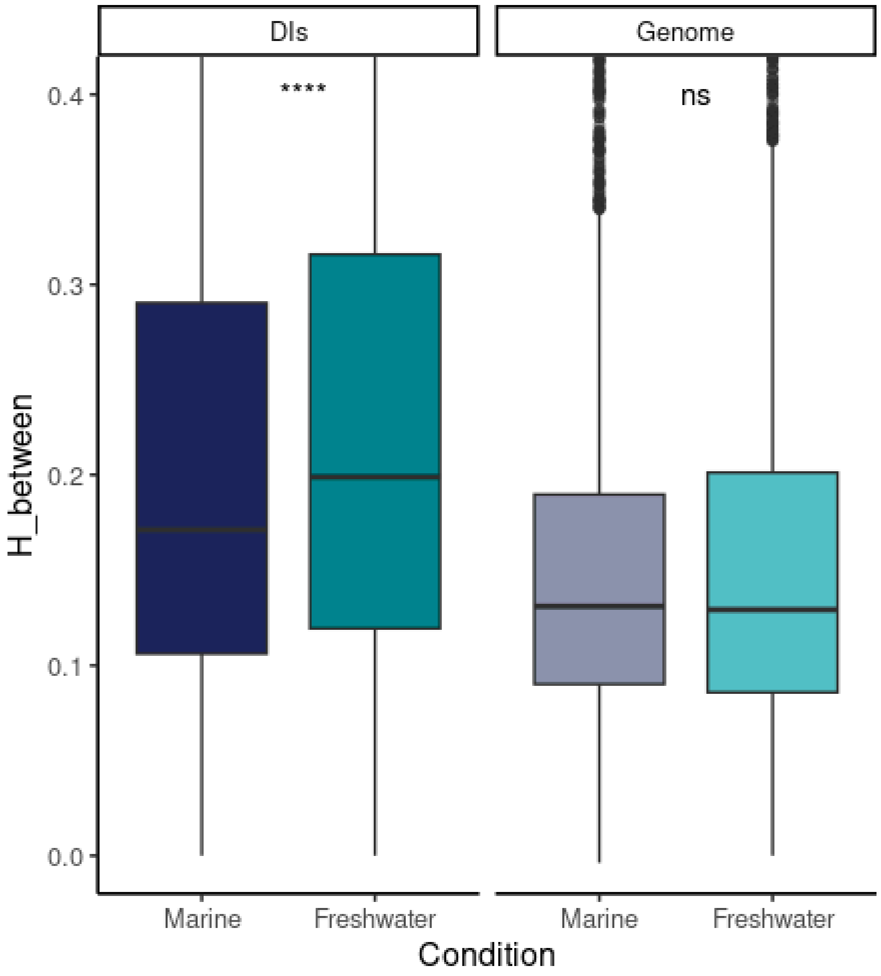

Figure S7. Genome-wide differences in inter-sample methylation entropy and differences within divergence islands (DIs); 6-CpG windows.

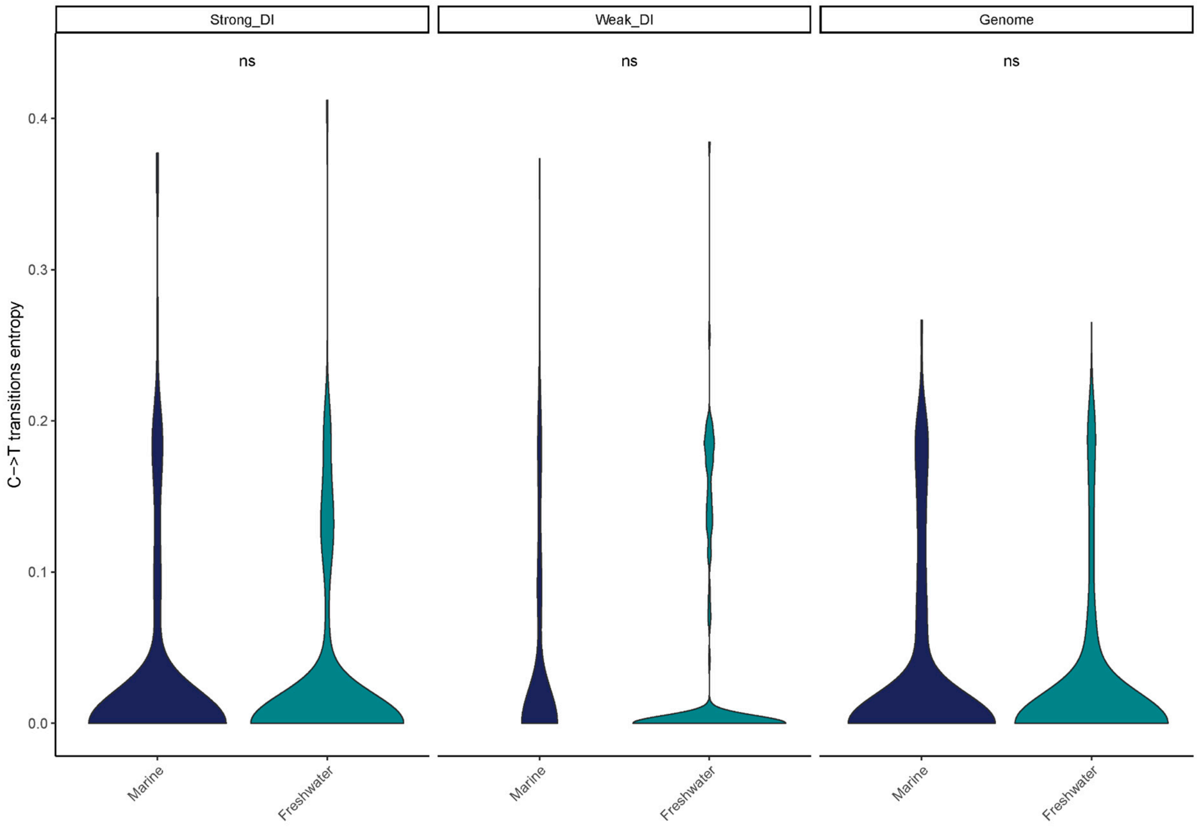

Figure S8. Genome-wide differences in C->T transitions entropy and differences within divergence islands (DIs).

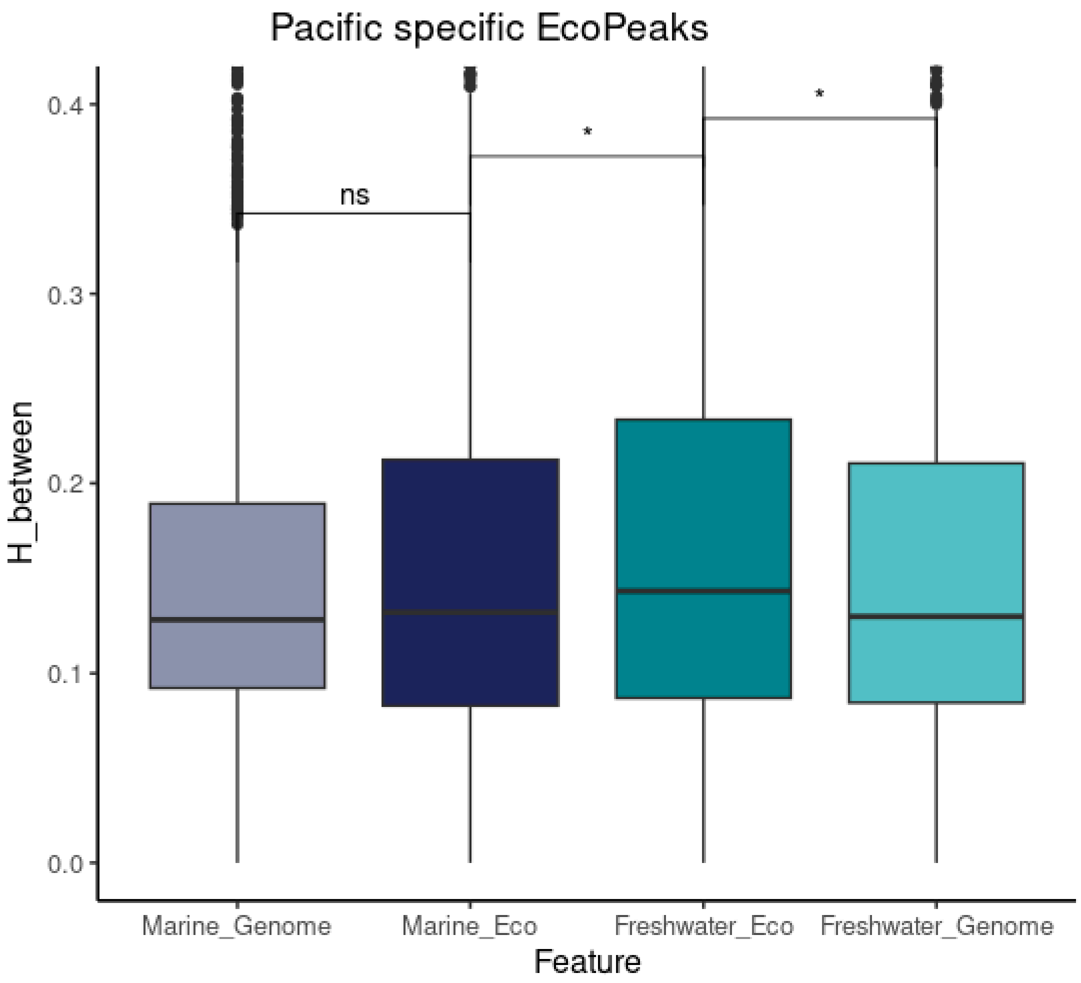

Figure S9. Genome-wide differences in inter-sample methylation entropy and differences within Pacific specific EcoPeaks.

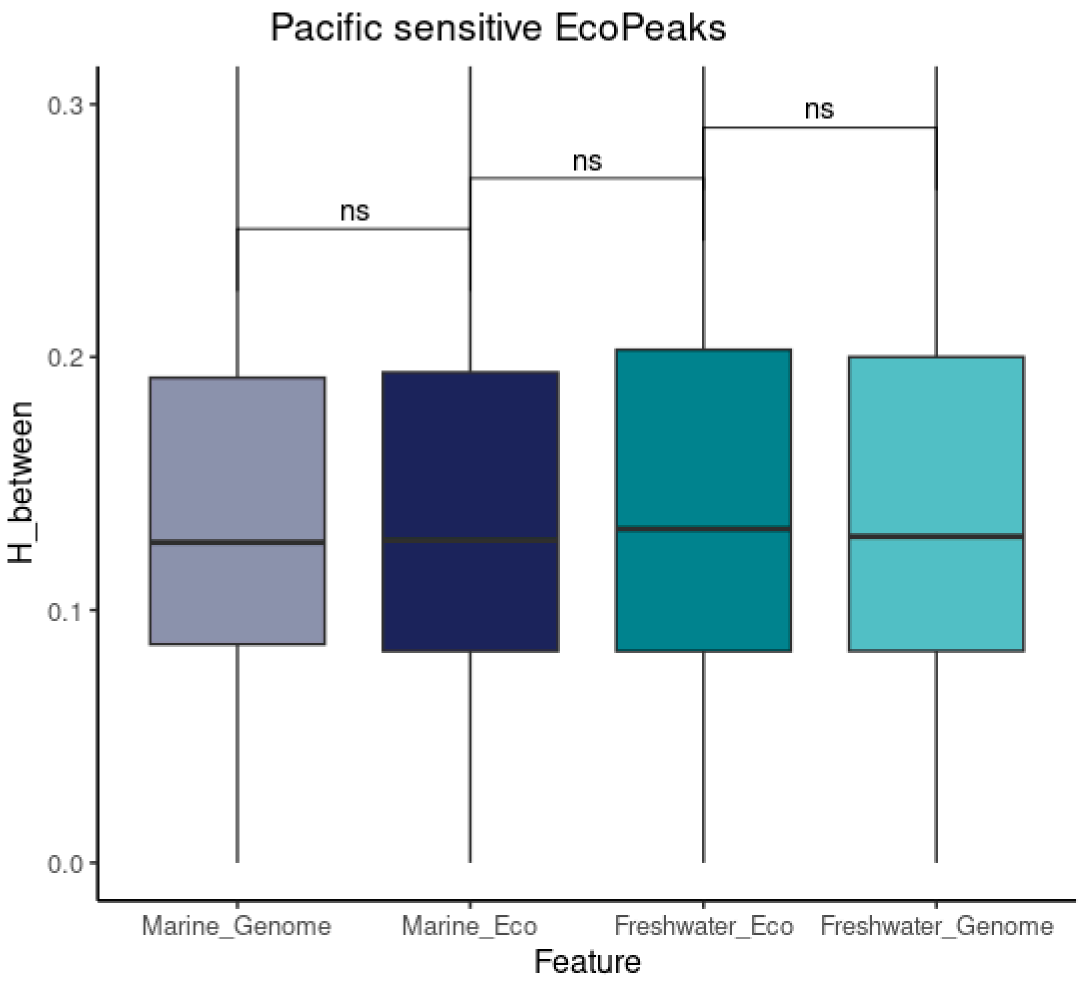

Figure S10. Genome-wide differences in inter-sample methylation entropy and differences within Pacific sensitive EcoPeaks.

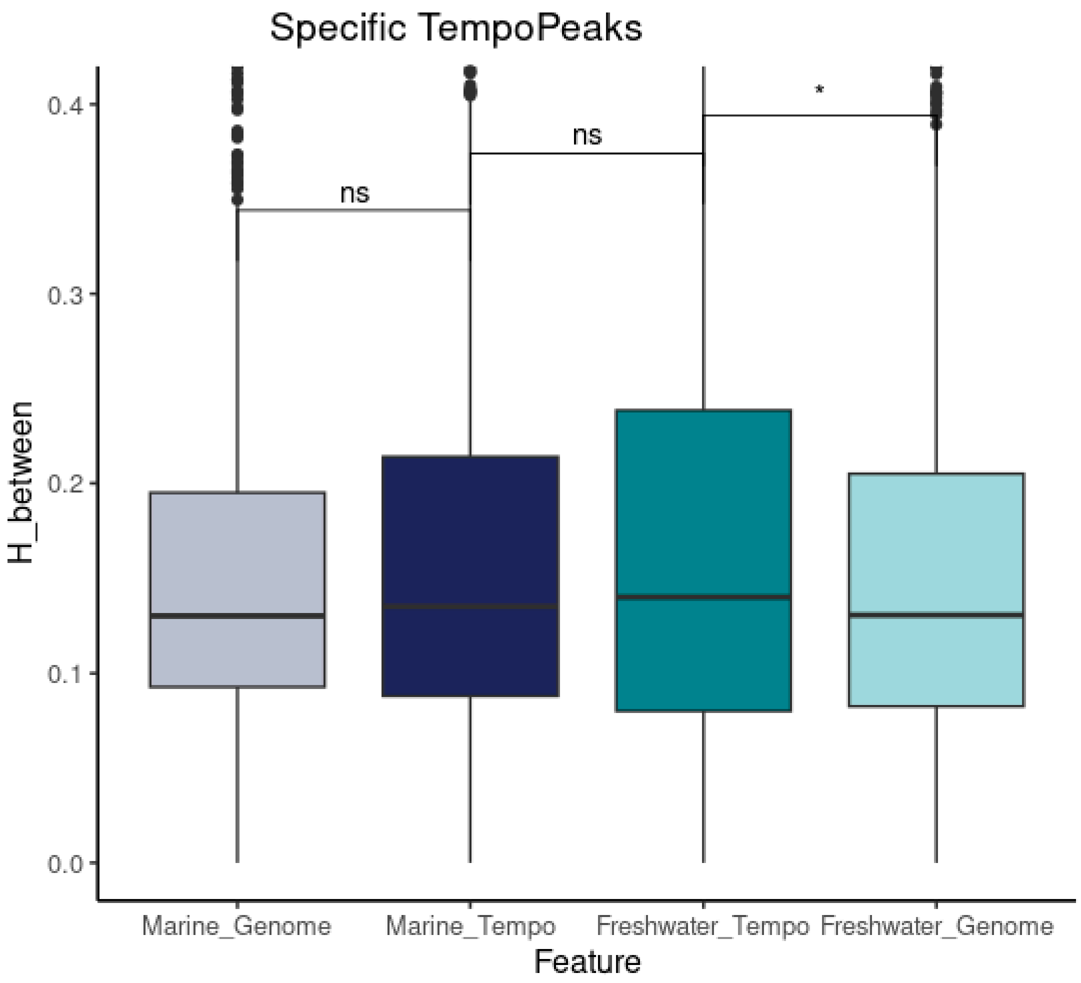

Figure S11. Genome-wide differences in inter-sample methylation entropy and differences within specific TempoPeaks.

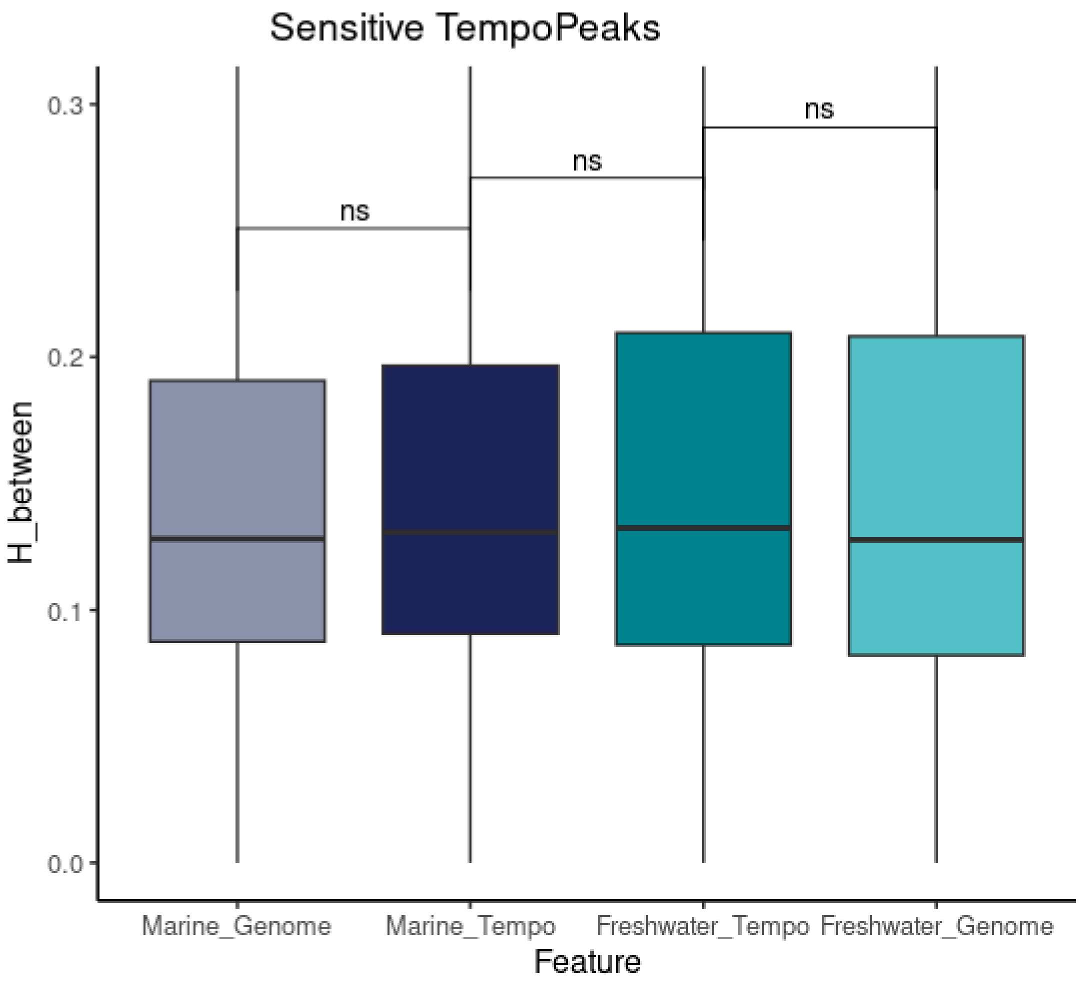

Figure S12. Genome-wide differences in inter-sample methylation entropy and differences within sensitive TempoPeaks.

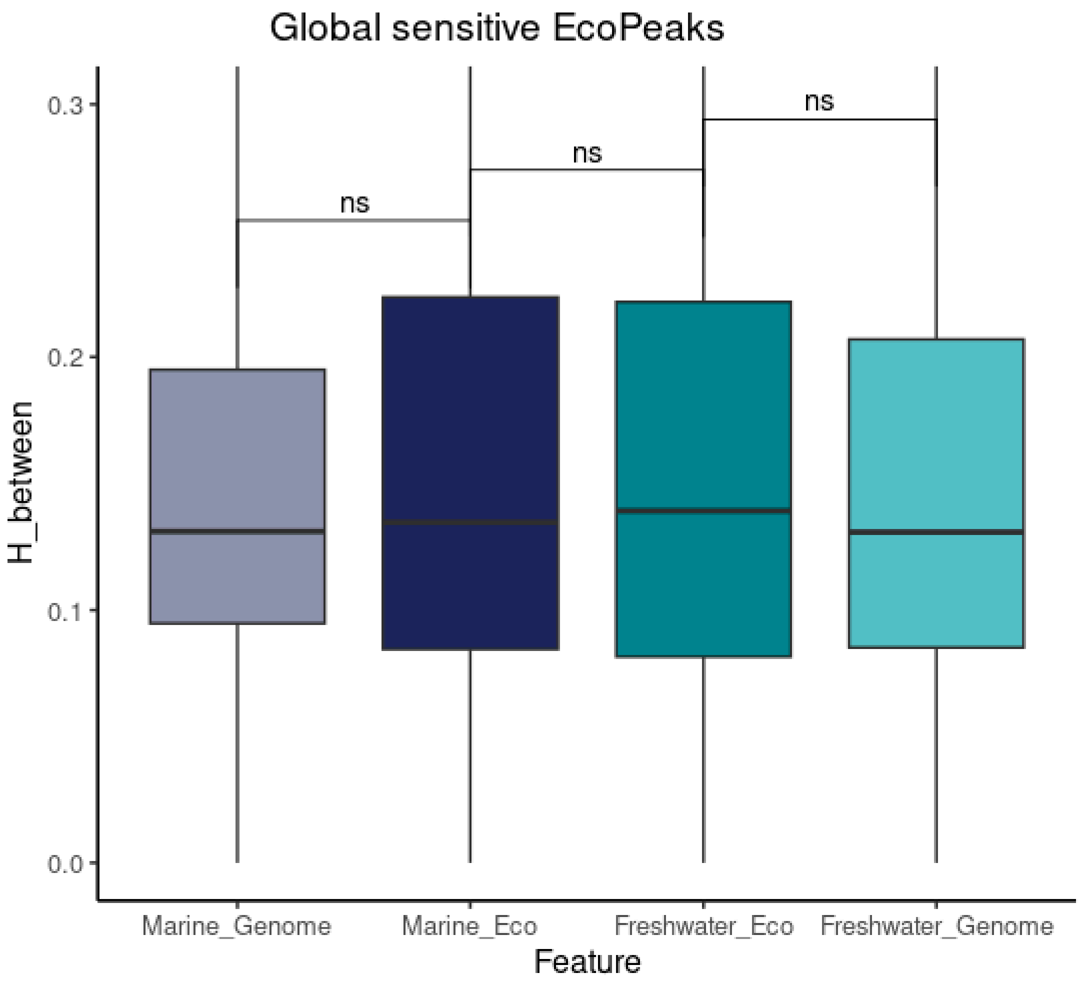

Figure S13. Genome-wide differences in inter-sample methylation entropy and differences within Global sensitive EcoPeaks.

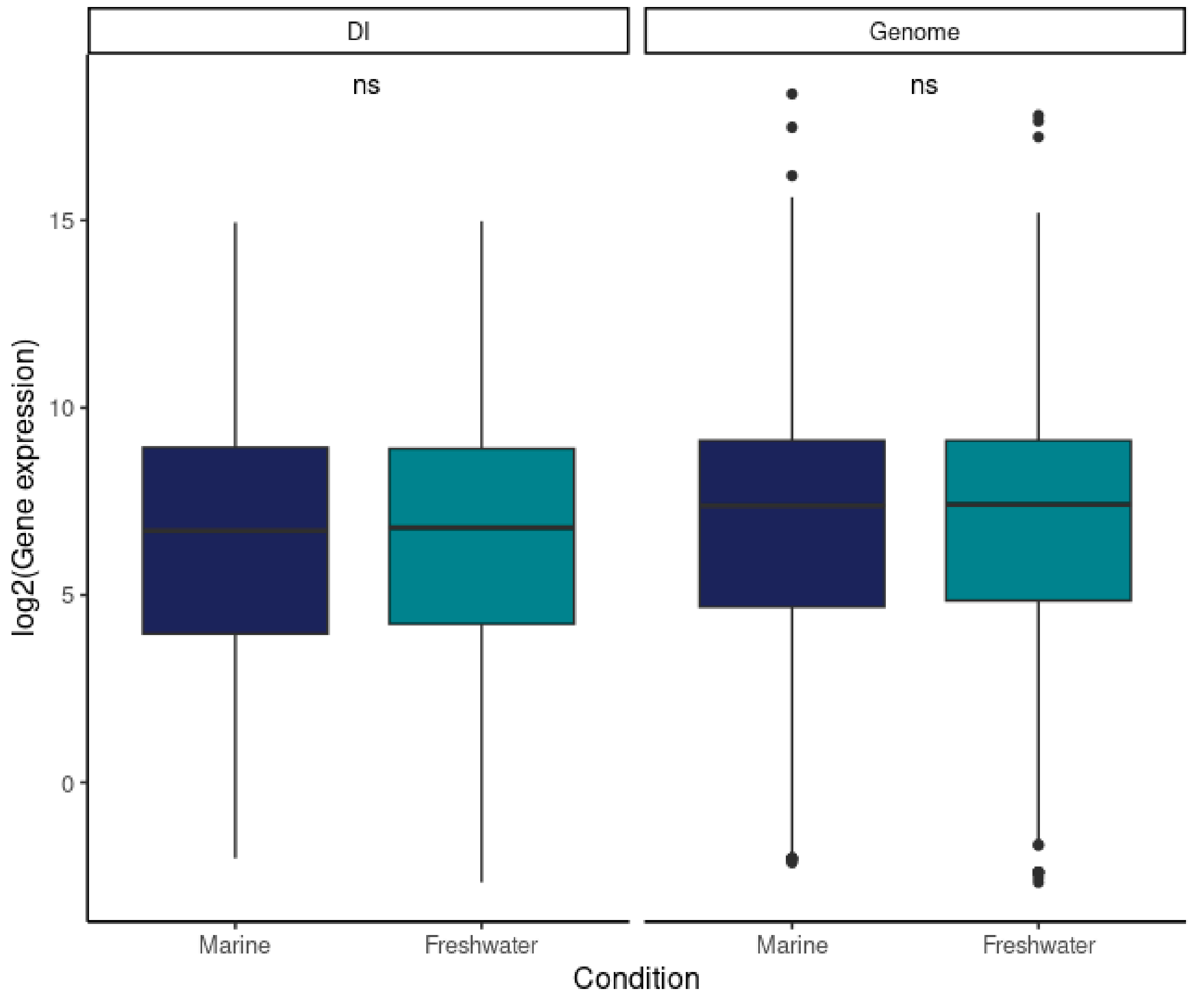

Figure S14. Genome-wide differences in gene expression and differences within divergence islands (DIs). Statistical comparisons based on normalized counts; values are log2-transformed for visual representation.

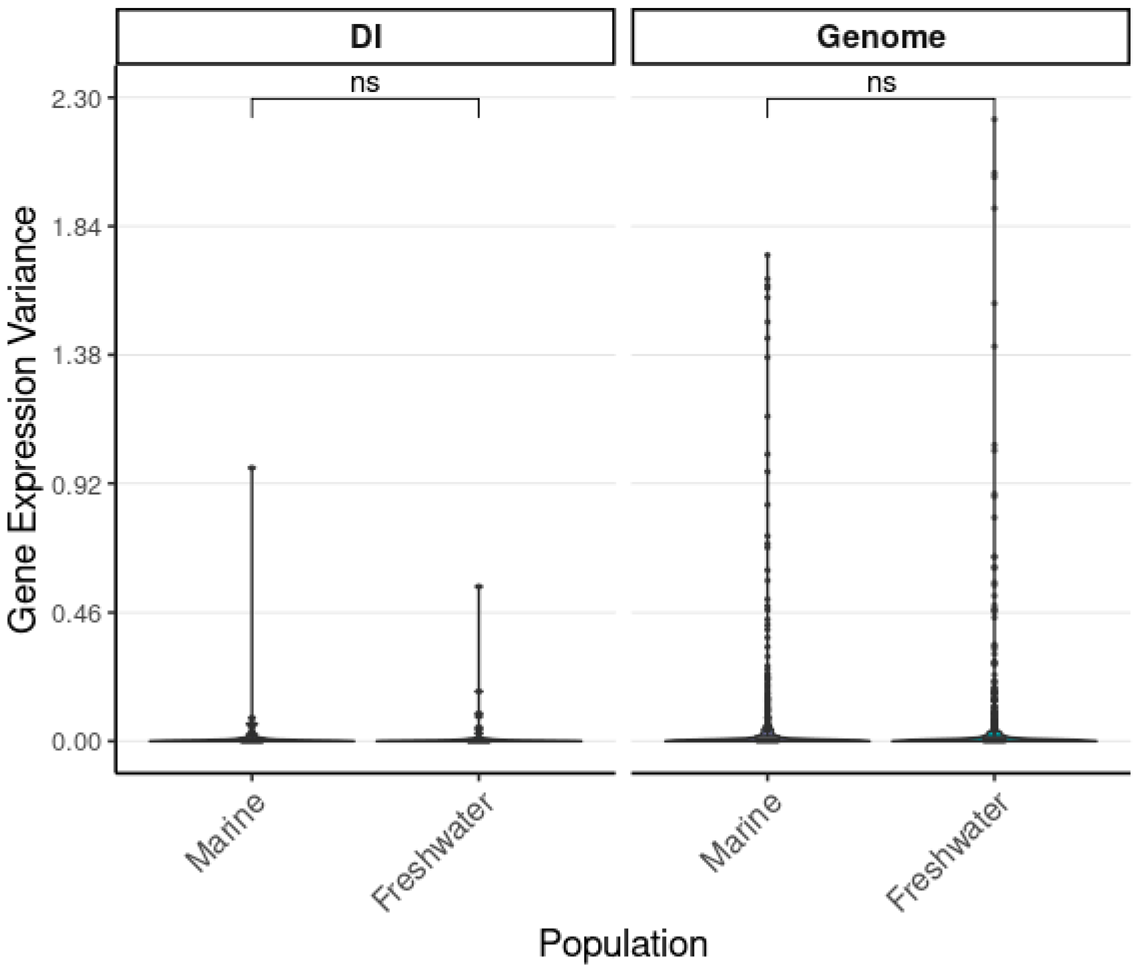

Figure S15. Genome-wide differences in gene expression variance and differences within divergence islands (DIs); scRNA-seq.

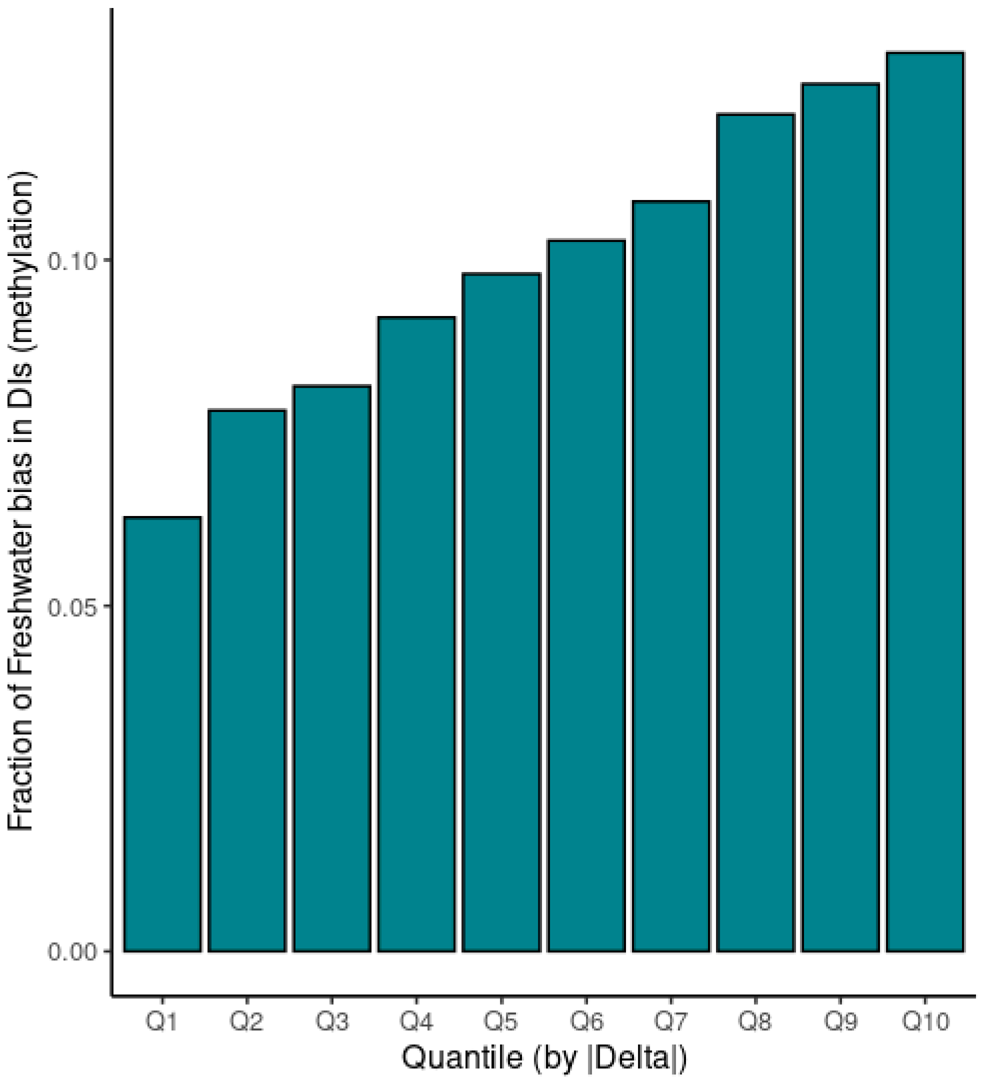

Figure S16. Fraction of DIs with freshwater bias (Δ = Freshwater − Marine > 0) in each quantile of absolute DNA methylation differences |Δ|. Quantiles are calculated across all data.

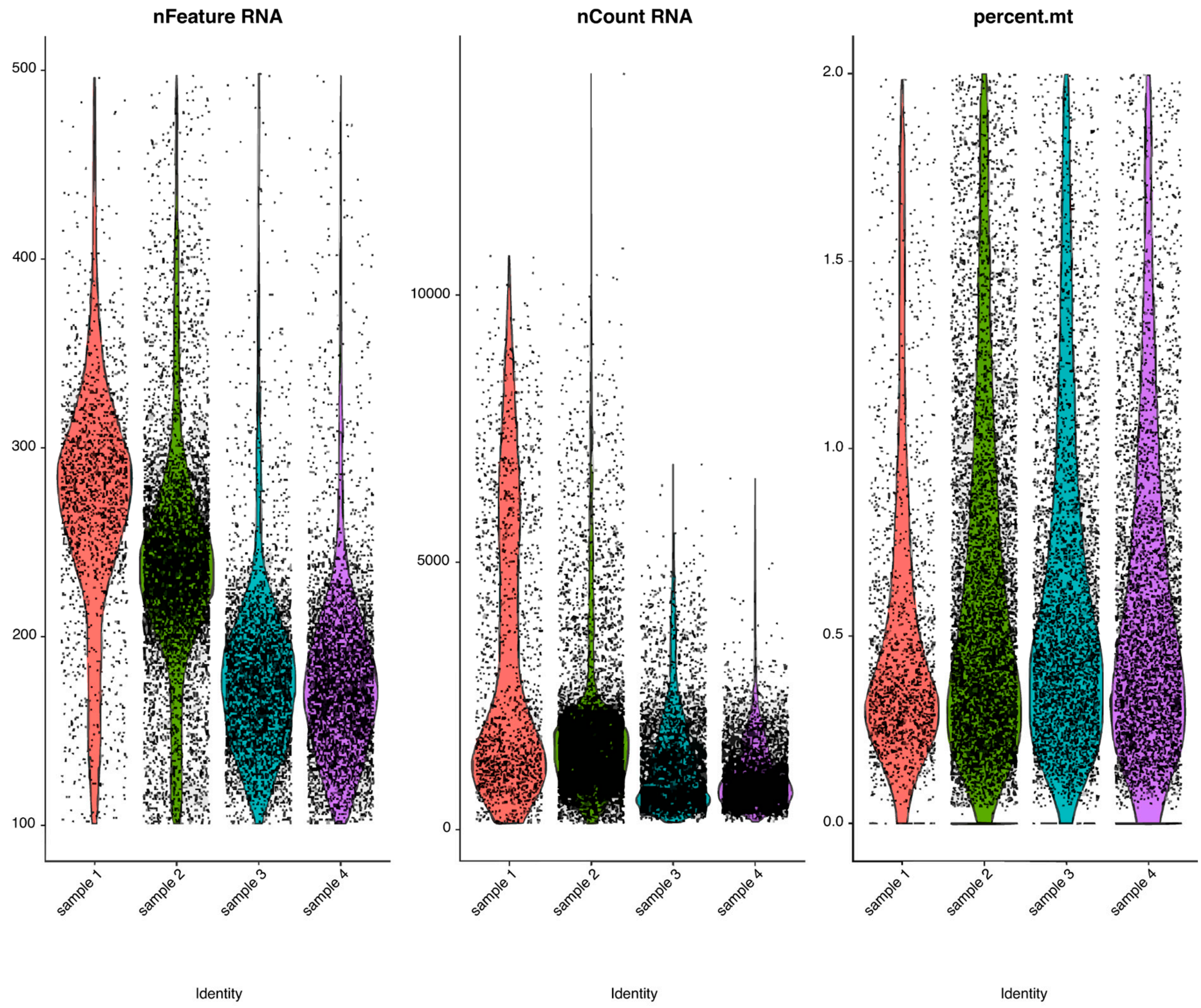

Figure S17. Quality control metrics for scRNA-seq data made in Seurat pipeline. Groups represent samples. Filtering thresholds: nFeature_RNA > 100, percent.mt < 2.

Supplementary Tables

Table S1. Enrichment analysis of DI-associated genes. Human orthologs were analyzed using rGREAT with the msigdb:C3 regulatory motif database.

Table S2. Nuclei-targeted and stock concentrations during isolation of nuclei for the scATAC-seq protocol.

Table S3. WGBS sample metadata and read mapping statistics.

Table S4. Cell Ranger run summary for the scRNA-seq dataset.

Table S5. Cell Ranger run summary for the scATAC-seq dataset.

Table S6. Parameters for QC filtration steps in Signac pipeline for each scATAC-seq sample.
